# Overview of Food Preservation and Traceability Technology in the Smart Cold Chain System

**DOI:** 10.3390/foods12152881

**Published:** 2023-07-29

**Authors:** Lin Bai, Minghao Liu, Ying Sun

**Affiliations:** School of Light Industry, College of Chemistry and Materials Engineering, Beijing Technology and Business University, Beijing 100048, China; 2230402043@st.btbu.edu.cn (L.B.); 2230602061@st.btbu.edu.cn (M.L.)

**Keywords:** cold chain system, food preservation, digital simulation, intelligent agriculture

## Abstract

According to estimates by the Food and Agriculture Organization of the United Nations (FAO), about a third of all food produced for human consumption in the world is lost or wasted—approximately 1.3 billion tons. Among this, the amount lost during the storage stage is about 15–20% for vegetables and 10–15% for fruits. It is 5–10% for vegetables and fruits during the distribution stage, resulting in a large amount of resource waste and economic losses. At the same time, the global population affected by hunger has reached 828 million, exceeding one-tenth of the total global population. The improvement of the cold chain system will effectively reduce the amount of waste and loss of food during the storage and transportation stages. Firstly, this paper summarizes the concept and development status of traditional preservation technology; environmental parameter sensor components related to fruit and vegetable spoilage in the intelligent cold chain system; the data transmission and processing technology of the intelligent cold chain system, including wireless network communication technology (WI-FI) and cellular mobile communication; short-range communication technology, and the low-power, wide-area network (LPWAN). The smart cold chain system is regulated and optimized through the Internet of Things, blockchain, and digital twin technology to achieve the sustainable development of smart agriculture. The deep integration of artificial intelligence and traditional preservation technology provides new ideas and solutions for the problem of food waste in the world. However, the lack of general standards and the high cost of the intelligent cold chain system are obstacles to the development of the intelligent cold chain system. Governments and researchers at all levels should strive to highly integrate cold chain systems with artificial intelligence technology, establish relevant regulations and standards for cold chain technology, and actively promote development toward intelligence, standardization, and technology.

## 1. Introduction

With the improvement of human living standards, people gradually begin to value the nutrition, quality, and variety of food. The demand for vegetables, fruits, meat, seafood, and other products is increasing, and the market prospect is considerable. However, equally, food is at risk of corruption and spoilage, which leads to great food waste and economic loss. Food loss is “a reduction in the quality of human edible food throughout the supply chain”, while food waste is “the loss of food that occurs at the end of the food chain” [1]. According to statistics from the Food and Agriculture Organization of the United Nations, up to 1.6 billion tons of food are wasted globally each year, with the edible portion reaching 1.3 billion tons [2,3]. In July 2022, five United Nations agencies jointly released the “2022 World Food Security and Nutrition Status” report, which stated that the global number of hungry people reached 828 million, exceeding one-tenth of the global total. About 258 million people in 58 countries and regions were affected by the severe food crisis, up from 193 million people in 53 countries and regions in 2021.

According to the European Union Statistics Office, the average person wasted 127 kg of food in 2020, 70 kg of which was produced by households. The per capita food waste of households is 70 kg, accounting for 55% of the total per capita food waste, and the remaining 45% is generated at other links of the food supply chain, among which food logistics and transportation waste accounts for a large proportion. According to data from the Food and Nutrition Development Research Institute of the Ministry of Agriculture and Rural Affairs, the average loss and waste rate of vegetables, fruits, aquatic products, grains, meat, milk, and eggs in China is as high as 22.7% each year, about 460 million tons, including 300 million tons of food loss in the production and circulation process. If the intelligent cold chain system is applied to food logistics transportation, it will greatly reduce food loss and waste in the food supply chain.

**Figure 1:**
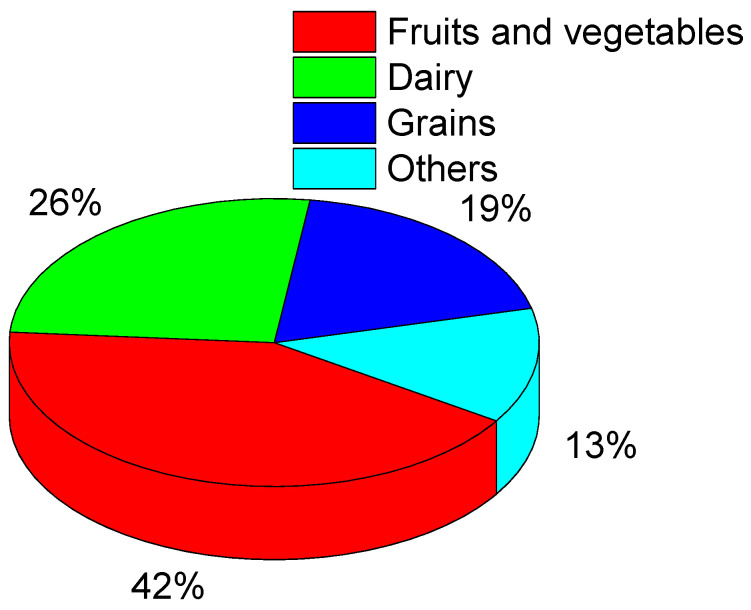
Percentage of different food waste.

According to statistics, there are approximately 1.3 billion tons of food waste produced worldwide every year [3]. Figure 1 shows the approximate distribution of all kinds of food waste [4], indicating that fruits and vegetables account for the largest proportion of food waste each year. This is because fruits and vegetables can still breathe after harvest, and unstable and inappropriate temperature and humidity increase the biological activity of microorganisms and accelerate their growth, leading to the increased spoilage and deterioration of food, which not only causes major economic losses and food waste but also increases the risk of food problems and foodborne diseases, thereby endangering human health [5].

Secondly, a major reason for food spoilage is packaging. Food packaging materials refers to materials such as plastic, fiber, glass, etc., used for packaging and holding food. Food packaging can prevent food products from leaking or breaking, and protects food from adverse effects from the external environment; it can also communicate with consumers through text or image information, providing greater convenience for consumers; and it can accommodate various sizes and shapes of products, making it easy to transport and handle. In addition, it can also have a protective effect, which can delay the spoilage of food to a certain extent [6]. Traditional packaging is designed to prevent fruit and other foods from being exposed to harmful environments, but currently, this is far from enough. With the continuous improvement of people′s pursuit of food quality and type, the requirements for food packaging are increasing. While achieving the goal of preventing environmental factors from contaminating food, it is also necessary to monitor food and keep it fresh at various stages, which ensures that the food can maintain the highest quality when it appears in front of people.

At present, the main preservation technologies for food include modified atmosphere preservation, preservative preservation, coating preservation, irradiation preservation, etc., which cannot monitor or adjust to environmental factors in real time. To improve this situation, researchers have developed intelligent cold chain systems and freshness sensors. Freshness sensors can be analyzed according to some specific environmental conditions or food indicators so as to achieve real-time monitoring of food freshness. On the other hand, researchers have combined smart cold chain systems with sensors and response systems to regulate various environmental conditions to extend food shelf life. There have been a lot of studies on freshness sensors and intelligent cold chain systems in the food industry. With the development of Internet of Things technology, many component systems are connected to carry out real-time data exchange and processing; optimize food quality parameters in the storage and distribution process; respond to environmental changes in the cold chain in a timely manner; and reduce losses and waste in each supply chain, thus achieving the integrity and precise control of the intelligent cold chain system. According to reports, the total demand for cold chain logistics in China in 2020 was 265 million tons, a year-on-year increase of 13.69%. Under the situation of pandemic prevention and control, the development momentum of cold chain logistics in 2021 was strong, with the total market demand exceeding 270 million tons.

**Figure 2:**
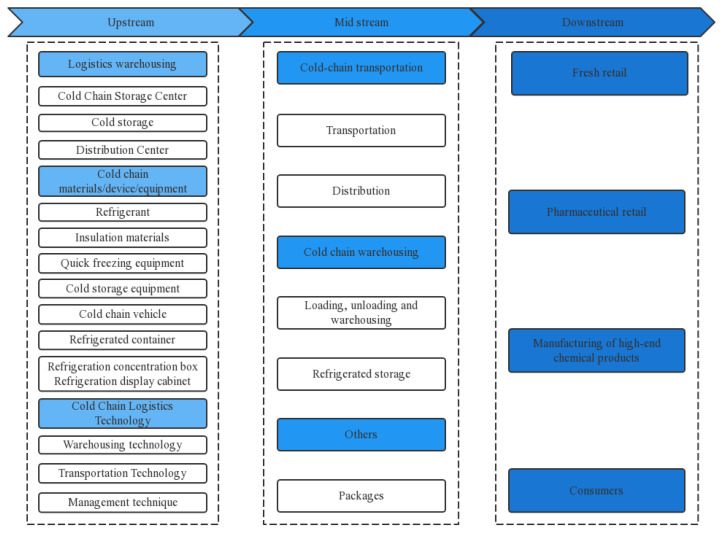
Panorama of cold chain logistics industry chain.

Intelligent cold chain systems can provide a suitable temperature and humidity for food production, processing, transportation, storage, sales, etc., and can effectively inhibit microbial activity and slow down the speed of food spoilage, thus reducing the risk of foodborne diseases. The intelligent cold chain system has been widely applied in the field of milk and dairy product preservation. For example, Zhang et al. [7] used the ISM method to analyze the hierarchical relationships between various factors of customers’ satisfaction with dairy products, and based on customer orientation, constructed a dairy cold chain logistics system. Secondly, in the field of fruits and vegetables, intelligent cold chain systems also have a large amount of applications. Su took subtropical fruits and vegetables from Guangxi as an example to study cold chain logistics in the context of Big data. They affirmed the advantages and importance of intelligent cold chain systems, and looked forward to the future development of the cold chain systems from three aspects: building a platform, building a new cold chain logistics mode, and strengthening management [8]. In the field of meat preservation, Liu used cold fresh lamb Xinjiang as an example to study its cold chain transportation monitoring system. They tested the freshness indicators of cold fresh lamb, introduced a cold fresh lamb monitoring system, and finally conducted a cold chain transportation simulation experiment on the cold fresh lamb, which highlighted the huge prospects of the cold chain system in meat preservation [9]. In intelligent cold chain systems, the integrity and closure of the cold chain should be realized, so as to avoid the large amount of food waste and excessive energy consumption. All links of the cold chain should be interrelated, and the upstream and downstream links cannot be separated to ensure the integrity and the closure of the cold chain. Figure 2 shows a panorama of the cold chain logistics industry chain.

Despite the rapid development of technology and its great potential in reducing food loss and waste, real-time monitoring technology based on the Internet of Things still faces many challenges. For example, there has been much research on real-time tracking and monitoring of environmental parameters using Internet of Things technology, but there is a lack of comparisons of specific environmental parameters of different foods and the selection of specific supply chain parameters. Additionally, the relationship between quality changes and environmental parameters cannot be established. As for fruits and vegetables, different products have different corruption dynamics and different environmental parameters related to preservation. Therefore, it is necessary to conduct real-time monitoring of various indicators of fruits and vegetables, and select different technologies that are based on a large amount of data to regulate different products.

This paper aims to combine traditional preservation technologies with artificial intelligence technologies, so as to develop a form of sustainable smart agriculture and guide fruit and vegetable preservation in order to reduce the waste of food resources and economic loss.

## 2. Influencing Factors and Preservation Technology of Fruit and Vegetable Shelf Life

Fruits and vegetables are essential foods in people′s daily life. Fruits and vegetables contain abundant vitamins, plant fiber, iron, selenium, and other essential elements to maintain human health. Additionally, they are low in calories, which can prevent some diseases such as obesity, hypertension, and hyperlipidemia. The rich anti-inflammatory and antioxidant components in fruits and vegetables have been proven to prevent depression and anxiety in people, and also reduce the risk of several chronic diseases such as heart disease, type 2 diabetes, and certain types of cancer [10,11]. However, fruit and vegetable products with high moisture content are extremely susceptible to decay. In order to extend their shelf life, we must understand their decay mechanisms and the factors influencing their corruption. Among them, there are many factors affecting the corruption of fruits and vegetables, mainly including respiration, transpiration, damage to appearance, and microbial action.

In order to extend the shelf life of fruits and vegetables, there are currently many effective preservation methods, including low-temperature preservation, modified atmosphere preservation, irradiation preservation, degradable film preservation, ozone preservation, etc.

### 2.1. Low-Temperature Preservation

Low-temperature preservation technology is common in daily life. By lowering the storage temperature, the respiration rate of fruits and vegetables will be reduced. At the same time, low temperature will also reduce the metabolic rate and water loss rate of fruits and vegetables, leading to a decrease in microbial activity and thus affect chemical reactions. Low-temperature preservation technology can improve the appearance and quality of fruits and vegetables to a certain extent, but if the temperature is too low, it may cause frostbite [12]. Therefore, the temperature is generally set at 0.5–1 °C above the temperature of the cold damage point of fruits and vegetables, and the relative humidity is kept at about 90–98%. Lowering the temperature can ensure that the enzyme activity of fruits and vegetables can be reduced without natural chilling injury, and the respiration rate of fruits and vegetables during storage can be inhibited so that the fruits and vegetables can enter an incomplete dormant state. Moreover, the low-temperature environment with high humidity can effectively inhibit the internal water loss of fruits and vegetables. Wang et al. found that low-temperature preservation is an effective way to maintain the tissue structure of fruit epidermis and pulp. Under low-temperature preservation conditions of −1 ℃, 1 μL/L 1-methylcyclopropene treatment can effectively inhibit ethylene production and respiratory rate,, inhibit the loss of soluble solids in hawthorn fruits, and decrease the quality loss rate and browning rate of fruits [13]. Zhao et al. [14] tested the soluble sugar content and metabolic level of nectarines at different temperatures to explore the factors affecting the shelf life of nectarine fruits. The results showed that fruits stored at near-freezing temperature (−1.4 ± 0.1 ℃) (NFT) showed no symptoms of freezing damage, and fruits stored at NFT exhibited a longer shelf life than those stored at 0 ℃ and 5 ℃. This method has been widely used in fruit and vegetable storage and cold chain logistics transportation. It is usually combined with other preservation technologies. For example, Wu et al. [15] explored the optimal preservation conditions of lotus roots, combined with heat treatment at 65 ℃ for 5 min, and then stored in preservative (0.3% CaCl_2_, 0.25% vitamin C, 0.3% citric acid) at −0.7–0 ℃. This process effectively inhibited browning, decay rate, polyphenol oxidase (PPO) activity, and sugar loss, while maintaining the hardness of lotus roots. Liu et al. [16] compared near-freezing point storage of apricot fruits with traditional low-temperature storage, and the results showed that near-freezing point storage was an effective method to maintain the apricot fruits’ flavor while extending their shelf life. These studies indicated that low-temperature storage is a relatively mature preservation technology at present, which is often used in combination with other preservation technologies to improve the preservation effect.

### 2.2. Modified Atmosphere Packaging

Modified atmosphere packaging means that, under certain storage conditions such as humidity and temperature, the metabolic rate of fruits and vegetables can be reduced and their storage period can be prolonged by regulating the concentrations of CO_2_ and O_2_. MAP (Modified Atmosphere Packaging), which is widely used at present, uses composite films to package fruits and vegetables, and establishes specific CO_2_ and O_2_ concentrations within the packaging of specific fruits and vegetables to inhibit the respiration rate of fruits and vegetables, so as to maintain their freshness. As a safe, pollution-free, and low-cost preservation technology, modified atmosphere packaging can maintain freshness in fruits and vegetables for 5–14 days, and currently has broad development prospects in China. Xing et al. [17] determined the change in quality of sweet cherries by setting the gradient concentration of CO_2_ at 0%, 5%, 10%, 15%, 20%, and 25% (O_2_ was 5% and the rest was filled with N_2_). The results showed that all the experimental groups had a certain inhibiting effect on the deterioration of sweet cherries. In particular, 2% CO_2_ concentration was the most effective in reducing the decay rate, maintaining hardness, and reducing the activities of polyphenol oxidase and peroxidase. Additionally, they still had good hardness, nutritional value, and taste after storage for 10 days. Ali et al. [18] found that the use of MAP storage at low-temperature (5 ± 1 ℃) can effectively inhibit the activities of polyphenol oxidase and peroxidase in litchi, delay surface browning, and maintain the good quality. The gas permeability partially depends on the thickness of the packaging materials. The decrease in thickness can improve the permeability of the film, but its strength will be reduced accordingly, which makes it difficult to meet the requirements of commodity storage and transportation [19]. Therefore, in order to optimize the technology, researchers have adopted microporous modified atmosphere packaging to achieve the coexistence of mechanical strength and freshness-maintaining properties. On the basis of effectively preventing anaerobic respiration of fresh food and the condensation of water vapor in the film, micropores help to regulate the relative contents of CO_2_ and O_2_ and the relative humidity inside the packaging for specific products [20].

### 2.3. Irradiation Preservation

Irradiation preservation involves directly irradiating fruits and vegetables with extremely-short-wavelength high-energy rays (such as γ rays, high-energy particle beams, infrared rays, ultraviolet rays, etc.) [21]. This technology can not only directly affect the molecular structure of microorganisms and the activities of enzymes and proteins in their bodies, inhibiting microbial activity, but can also inhibit the respiration rate of fruits and vegetables and prolong their maturity. Thus, the storage period of fruits and vegetables is prolonged, and the flavor and safety of fruits and vegetables are not affected [22]. Irradiation preservation itself belongs to the category of “non-thermal” processing technologies, with a good sterilization effect, no chemical residue, simple operation, safety, environmental protection, and other advantages. The irradiation effect will be affected by irradiation intensity and irradiation time. Ruan et al. used UV irradiation to preserve mangoes, and their results showed that UV irradiation significantly prolonged the shelf life of mangoes compared with the control group. Maurer et al. irradiated grapes with ultraviolet light, and the results showed that compared with the control group, the antioxidant oxidase activity, total phenol content, and anthocyanin content of grapes irradiated with ultraviolet light were all improved, and the quality of samples in the experimental group was higher than that of the control group after 5 days of storage [23]. Araque et al. [24] evaluated the effect of repeated short dose ultraviolet radiation on strawberry quality. The results showed that, compared to traditional single-high-dose pre-storage irradiation, the repeated low dose of UV radiation more effectively reduced the changes in strawberry, and it further reduced the amount of mold and yeast to a greater extent. However, when using irradiation preservation technology to preserve fruits and vegetables, it is necessary to consider the potential threat of this technology to human health. For example, when irradiating foods using γ rays, it is necessary to design thick walls for the site to prevent radiation leakage. During the processing, it is also necessary to consider the hazards of the radiation to which workers are directly exposed. Although irradiation preservation technology has been proven to have no negative impact on food quality or human health within the dose range, consumers’ acceptance of irradiated food is relatively low [25]. According to EU regulations, any irradiated food must be clearly labeled as “irradiated” or “treated with ionizing radiation”, making it easy for consumers to see clearly and be fully aware of the food they purchase.

### 2.4. Nanotechnology Preservation

Nanotechnology preservation has long-term sterilization and preservative abilities. It works by filling nanoscale antibacterial agents into the packaging material. Commonly used antibacterial agents include nano-Ag, nano-ZnO, nano-TiO_2_, etc. They have antibacterial and antiviral properties, have low oxygen permeability, act as a barrier to carbon dioxide, and have other characteristics, so they play an indispensable role in fruit and vegetable preservation. However, some nanoparticles will remain in the human body, posing a potential threat to human health. Guan et al.[26] combined CuO nanoparticles and ZnO nanoparticles into sodium alginate chitosan using the layer-by-layer method to prepare a double-layer film with antibacterial properties. The research results showed that the films had good mechanical, barrier, optical, and biodegradable properties. They had excellent antibacterial ability against Escherichia coli and Staphylococcus aureus. Duan et al. [27] prepared a composite film containing konjac glucomannan (Konjac Glucoman-KGM), k-carrageenan, and TiO_2_ nanoparticles and tested its preservation effect on strawberries, and the results showed that the film showed good photocatalytic antifungal activities, which could effectively prolong the storage time of strawberries. Table 1 shows the application of nano-antibacterial agents under the current preservation technology in food preservation.

However, this technology may leak Ag^+^, Ti^2+^, etc. into the fruits, vegetables, and external environment, which is controversial for human health and environmental safety. Therefore, although nanotechnology is a new and effective preservation technology, the safety evaluation and the development of pollution-free nanomaterials still needs to be further studied and perfected.

### 2.5. Other Preservation Technologies

Ozone treatment has also been used for fruit and vegetable preservation in recent years. Its principle is that, when oxygen is exposed to ultraviolet radiation, it will split into a single oxygen or primary oxygen, then react with oxygen and generate ozone. Through the strong oxidation of ozone, it can be used for sterilization, disinfection, deodorization, and preservation in cold storage. This technology takes advantage of the instability of ozone itself, and it is more beneficial to apply it to assist preservation in cold storage, because the final product of its decomposition is oxygen, which will not leave any harmful residue on fruits and vegetables. Ozone has three mechanisms of action in cold storage: one is killing microorganisms, disinfection, and sterilization; the second is to remove organic smells through inorganic oxidation deodorization; thirdly, the products of metabolism are oxidized, thus inhibiting the metabolic process. This method also plays an important role in the field of quality preservation and maintaining freshness. Ozone preservation can prolong the shelf life of fruits and vegetables by 3–10 times. Ozone has been recognized as a generally recognized as safe (GRAS) substance by the United States Food and Drug Administration (FDA). This technology was also combined with irradiation preservation to test its preservation effect on cabbage, and it was found that it could effectively extend the preservation period of cabbage without causing any damage to it [42].

Biodegradable film preservation refers to the use of biodegradable polymer materials to make films for the preservation of fruits and vegetables. The materials can degrade quickly in the natural environment without causing pollution to the environment. This method is worth popularizing and applying on a large scale in today′s increasingly severe environmental situation. The representative degradable materials are polylactic acid (PLA), polybutylene terephthalate (PBAT), polybutylene succinate (PBS), polyvinyl alcohol (PVA), gelatin, and other materials. This technology can also be combined with modified atmosphere packaging technology, which not only increases the preservation effect, but also allows the packaging substrate to naturally degrade. This method reduces the environmental pressure, and has broad development prospects.

## 3. Intelligent Cold Chain System

The intelligent cold chain systems ensure food quality and safety through the comprehensive real-time monitoring of food status in the food supply chain. The systems organically combine the sensors with Internet of Things technology. The sensors monitor the temperature, humidity, and the operating state of refrigeration equipment in the area where the food is located in real time. The data are transmitted to the data processing module in real time by using wireless communication technology. After processing and analyzing the data, the data are transmitted to the server and client. The server can conduct early warning and regulations according to the data analysis results, and the client can conduct real-time queries of food logistics information, food status, and food history information. To achieve the integrity of cold chain systems, the sensors and their data transmission are indispensable [43,44].

### 3.1. Freshness Sensor

**Figure 3:**
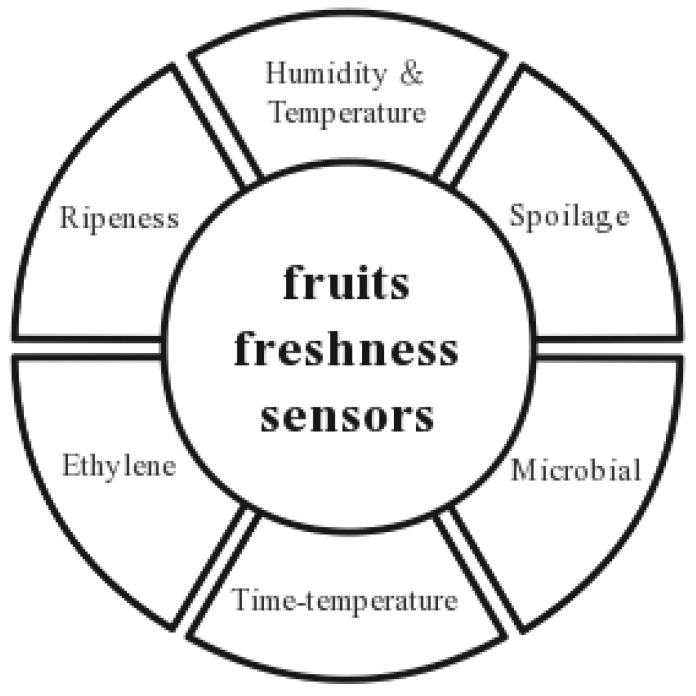
Freshness sensor classification.

Figure 3 shows the types of fruit and vegetable freshness sensors classified according to different environmental parameters.

At present, the commonly used sensors in cold chain systems include temperature sensors, humidity sensors, time–temperature sensors, and sensors based on radio frequency identification (RFID).

#### 3.1.1. Temperature Sensor

In the transportation process of fruits and vegetables, if the temperature is too high, the freshness of fruits and vegetables will change rapidly. Temperature fluctuations will lead to the phenomenon of water condensation in the environment where fruits and vegetables exist. The water condensation will promote the growth of fungi, bacteria and other microorganisms, also accelerating the corruption of fruits and vegetables. Secondly, water condensation may also fog the outer packaging of fruits and vegetables, which could affect consumers′ purchasing decisions. In cold chain logistics, the temperature sensors will carry out real-time monitoring of the environmental parameters around the area where the fruits and vegetables are, and regulate the temperature when it exceeds the threshold. For example, Zhang et al. [45] firstly developed a dual-mode freshness sensor that monitors both ammonia and temperature to monitor seafood freshness. The results indicated that the prepared ammonia vapor detection gas sensor exhibits highly recognizable fluorescence color changes, and the color changes from white to yellow as the storage temperature increases under natural light. Zhu et al. [46] monitored related indexes of fresh garlic quality such as temperature, relative humidity, gas composition, and ethylene through an information acquisition system based on wireless sensor networks. The results showed that the visualization of environmental parameter data enhanced the real-time perception of fresh garlics’ storage quality from producers to consumers, as well as the transparency of the supply processes and the level of cold chain management. De Oliveira et al. studied the effects of temperature and relative humidity on grape shelf life during transportation in cold chain trucks. Through multi-location and multi-sensor monitoring, it was verified that grapes of the same batch would have different qualities. Therefore, the principle of “first expired, first out” rather than “first in, first out” should be adopted as far as possible in the subsequent warehousing and discharging process [47].

#### 3.1.2. Humidity Sensor

Environmental humidity is also an important parameter that needs to be monitored. At present, capacitive sensors are widely used in humidity monitoring, which use the change in capacitance to monitor the change in humidity. The sensors consist of two parallel metal plates filled with a dielectric material between them, and the two parallel plates are connected to the voltmeter. When the humidity in the environment changes, the dielectric constant of the dielectric material between parallel plates changes, which causes the capacitance between parallel plates to change, resulting in a detectable voltage difference. The relative humidity in the environment is determined according to the following formula [48]:

(1) $$C=\frac{k\varepsilon_{0}A}{d}$$

where *C* is the capacitance, *A* is the area of the parallel electrode plate, $\varepsilon_{0}$ is the vacuum dielectric constant (8.85 × 10^−12^ F/m), *d* is the distance between the two plates, and *k* is the dielectric constant of the dielectric material between the two plates [48].

(2) $$V=\frac{Q}{C}$$

where *V* is the voltage, *Q* is the amount of charge on the plate, and *C* is the capacitance.

When the ambient humidity changes, the capacitance of the humidity sensors changes accordingly, that is, when the relative humidity increases, the humidity sensitive capacitance increases, and vice versa. The conversion circuit of the sensors converts the change in humidity sensitive capacitance into a change in voltage, corresponding to the change in relative humidity between 0 and 100% RH, and the output of the sensor is a linear variation of 0–1 V. For example, Pereria et al. [49] monitored changes in relative humidity of packaged foods which was packaged with a gelatin-based nano-ZnO composite film. The dielectric properties depend on moisture content, and the nano-ZnO layer acts as a semiconductor that provides a detectable signal based on the N-type semiconductor properties. The results indicated that the impedance of the composite film decreases with an increase in relative humidity, and it has different electrical responses in environments with different relative humidity. As the relative humidity values change from low to high, the film exhibits a rapid electrical response and good stability.

In addition, the accuracy of the sensors are also determined by relative humidity, such as ±2% RH in the medium and low humidity range (0–80% RH) and ±4% RH in the high humidity range (80–100% RH). Relative humidity is a function of temperature, which will have a serious impact on the relative humidity in the space. If the temperature varies by 0.1 ℃, the error of humidity change generates 0.5% RH. If the space cannot achieve constant temperature, it is difficult to obtain a highly accurate humidity measurement. Because the humidity will change with the change in temperature, the premise of humidity control is to control the temperature. Integrated temperature and humidity sensors are often used in daily life rather than humidity sensors. For example, Liu et al. [50] used multi-sensor technology (MST) to monitor the temperature, relative humidity, oxygen concentration (O_2_), carbon dioxide (CO_2_), ethylene (C_2_H_4_), and other parameters in the cold chain logistics of Korla fragrant pear so as to evaluate the cold chain logistics system. The experimental results indicate that the MST technology can improve the precision of sensors’ data acquisition. By analyzing various parameter data, Liu et al. found that the preservation quality of Korla fragrant pear can be effectively improved.

In most cases, if there is no precise means of temperature control available or if the space being measured is unsealed, an accuracy of ±5% RH is sufficient. For the cold chain systems which require accurate control of constant temperature and humidity, it is necessary to track and record humidity changes at any time. Generally, humidity sensors with an accuracy of ±3% RH or higher are selected.

#### 3.1.3. Time–Temperature Sensor

The time–temperature sensors are designed according to certain time and temperature conditions and can record the change in time and temperature. The working principle is based on the different reactions of two or more substances during the process of time and temperature change, resulting in irreversible discoloration of the indicator [51]. This is represented by Arrhenius’s formula in physical chemistry:

(3) $$k=Ae^{-E_{a}/RT}$$

The chemical reaction rate constant *k* in the solution increases with the increase in temperature. When the space temperature exceeds the threshold, a chemical reaction takes place and is ultimately manifested by the color or state of the solution. At present, the more common time–temperature sensors can be roughly divided into two types. The first is the solution diffusion type: its working principle is based on Brownian motion, and the substance diffusion speed increases with the increase in temperature. The other is the pH discoloration reaction type, which can be divided into polymerization reaction type and enzyme reaction type. Temperature change leads to polymerization reactions and enzyme-catalyzed reactions, and the pH indicators undergo color changes. Regardless of the principle, the monitoring parameters of the time–temperature sensors are basically the same, and include the two parameters of time and temperature. The setting of threshold temperature should take into account the specific corruption mechanism of different fruits and vegetables. Currently, time–temperature sensors are more likely to be used in smart food packaging systems, allowing consumers to identify product quality more quickly and more accurately. For example, Choi et al. designed a time–temperature indicator (TTI) based on a self-healing nanofiber pad that is intelligent, damage-resistant, and sensitive. Due to light scattering induced by nanofibers, the mat is opaque during refrigeration, but self-healing-induced interfiber fusion is prone to occur at room temperature, thus making the mat irreversibly transparent and providing early warning and reporting, effectively ensuring the continuity of refrigeration in the food supply chains [52]. Wang et al. prepared photoluminescent chemical TTI using the principle of heat-induced fading in the reverse reaction of photoluminescent compounds. The reaction rate and fading degree change with the accumulation of time and temperature, which can directly indicate the change in food shelf life [53]. Yamamoto and Isshiki developed a time–temperature sensor based on the Maillard reaction for food temperature monitoring, and the color of the sensor changes with time and temperature. The results showed that the device is helpful to monitor and warn of the growth of microorganisms in chicken [54].

#### 3.1.4. Sensors Based on Radio Frequency Identification Technology (RFID)

Radio frequency identification (RFID) sensors. The RFID tag is an automatic identification technology that allows non-contact bidirectional radio frequency (RF) data communication. It can read and write to the recording medium (electronic tag or RF card), so it can identify targets and exchange data. RFID tags were originally designed to replace optical bar codes to achieve traceability of products in the food supply chains [48]. RFID tags can record the whole fruit and vegetable supply chain throughout the entire process, and also monitor multiple products and types of information, including various environmental parameters in the supply chains, product sources, and other information [55].

RFID tags are classified into passive tags and active tags. The main difference between the two types of tag is whether they have an internal power source; passive tags have no internal power source and rely on electromagnetic energy to function, while active tags have a power chip inside that can be self-powered, with which they can be traced. However, compared to passive tags, the high cost of active tags is currently a major issue that limits their large-scale application. Another major drawback of RFID tags is their readability when surrounded by products with high metal or moisture content, as well as the limitations in real-time data collection during transportation.

At present, researchers are trying to combine RFID tags with Internet of Things technology to carry out real-time monitoring of perishable foods such as fruits, vegetables, and meat during the process of production, processing, transportation, and storage, so as to ensure that the food remains fresh when presented to consumers. By combining it with wireless communication technology, real-time data collection and recording can be realized. These systems require RFID tags with antennas and RFID scanners. RFID tags use wireless communication technology to transmit information and data about products to the central network through antennas and scanners for data storage and processing. In addition, RFID scanners can read multiple tags simultaneously and support single piece and batch scanning. For example, Alfian et al. [56] not only reduced the corruption of meat during transportation, but also achieved the traceability, information recording, and the information inquiry for meat in whole supply chains and prevented the circulation of fake and shoddy products in the supply chains. Wu et al. proposed a cold chain system that integrates the Internet, radio frequency identification (RFID), and General Packet Wireless Service (GPRS) technologies to monitor and manage cold chain vehicles. The results showed that the RFID-based system had advantages, such as no human involvement, no visual tag reading, fast data exchange, and higher resistance to humidity and environmental conditions. Bhadra et al. used a wireless sensor to detect fish rot. The resonant frequency of the sensor varied with the total volatile alkali nitrogen content (TVB-N) in the top space of tilapia packaging. Tilapia was tested at 24 ℃ and 4 ℃, and the results showed that the sensor could effectively identify microbial information in tilapia at 24 ℃ and 4 ℃ storage conditions, indicating the potential of the sensor in real-time monitoring of fish spoilage [57].

Although RFID tags can monitor the environmental information and product information in cold chain systems, they cannot monitor the biochemical reactions involved in the process of food corruption, nor can they accurately monitor the change in food quality. Other food freshness sensors could be used to monitor food temperature, relative humidity, light, pressure, pH value, etc., combined with the environmental information monitored by RFID tags. Real-time monitoring of food quality and real-time prediction of remaining shelf life can be realized to provide important support for consumers′ purchase decisions [58]. In addition, the production cost of RFID tags is relatively high, which limits their application in fruit and vegetable products, and there is no unified international standard for the use of RFID tags. Therefore, reducing the production cost of RFID tags in the future is another direction we should explore.

### 3.2. Data Transmission and Processing Technology

To achieve the intelligence of the cold chain systems, the transmission and processing of data are very crucial. This requires the network layer to transmit and process the data in real time. Sensor devices adjust the relevant parameters of the cold chain systems by transmitting the collected data in real time to the network layer for analysis and processing, in order to achieve the optimal preservation effect.

#### 3.2.1. Wi-Fi and Cellular Mobile Communication

The transmission distance of Wi-Fi can reach 100 m and the cost is relatively low. However, Wi-Fi is easily interfered with by other devices using the same frequency band during its use, so the service quality of Wi-Fi is contingent to a certain extent [59]. However, the data transmission speed of Wi-Fi is much faster than that of other short-range communication technologies, and as a result, many devices in current cold chain systems use Wi-Fi connections [60]. In recent years, researchers have found that, while Wi-Fi has the advantage of high data transmission rate, it also has the disadvantage of high power consumption, which is not conducive to directly connecting to sensor devices that rely on battery power. Moreover, sensor devices are only used for environmental parameter monitoring and do not need high data transmission rates. Cellular communication technology’s high data transmission rate and high power consumption are similar to those of Wi-Fi communication, but it can support long-distance data transmission, which is an advantage over Wi-Fi connections [61]. At present, the rise of 5G technology provides great development prospects for the connection of equipment in the cold chain systems, and the goal of mass data transmission and low energy consumption may be realized by using 5G technology [59].

#### 3.2.2. Short-Range Communication Technology

Short-range communication technologies include near-field communication (NFC), radio frequency identification (RFID), Zigbee, and Bluetooth technologies. Their typical travel distances range from a few centimeters to a few hundred centimeters [62]. NFC uses a frequency band of 13.56 MHz, and the transmission distance is as short as 10 cm [63]. At present, almost all mobile phones have the function of reading NFC, so the prominent advantage of this technology is its low cost and easy integration [64]. The data transmission range of Zigbee and Bluetooth is not very different, reaching around 100 m [64], and their cost and power consumption are relatively low. The difference between the two is that Zigbee is preferred over Bluetooth when a large number of sensor devices need to be connected [65], but Bluetooth is superior to Zigbee in energy efficiency [63]. For RFID technology, passive tags have the advantages of low cost and low power consumption, and the data transmission distance is about 10 m, while active tags have stronger signals and a larger range, and the transmission distance can reach 100 m [66]. At present, the ability of RFID tags to withstand harsh conditions and be installed quickly has attracted the attention of researchers, who combine short-range with other wireless communication technologies to overcome the disadvantages of short transmission distances.

#### 3.2.3. Low-Power Wide-Area Network (LPWAN)

The low-power wide-area network is a network layer technology of the Internet of Things (IOT), which has appeared in recent years to meet the requirements of long-distance and low-power communication. It is characterized by a long transmission distance, generally more than 5 km. The low node power consumption, simple network structure, and low operation and maintenance cost are also advantages of LPWAN. The emergence of low-power wide-area network technology has laid the foundation for the larger-scale development of the Internet of Things.

Lora, Weightless, 802.11 ah, and NB-IoT are all types of low-power wide-area network technologies. LoRa uses unauthorized frequency bands, while NB-IoT uses authorized frequency bands, which makes the service quality of NB-IoT better than LoRa because it has no duty cycle constraints [61,67]. The main advantages of LoRa are its low cost of connecting terminal equipment and no operational cost of data transmission [68]. However, in practical application, the selection of specific technology also needs to consider specific products, the amount of equipment, transmission distance, coverage range, and so on.

## 4. Application of Traceability Systems

### 4.1. Traceability Based on Internet of Things Technology

**Figure 4:**
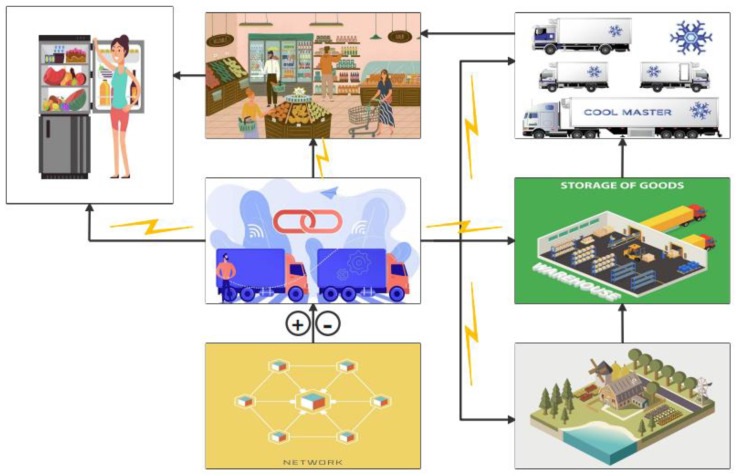
Example of IoT application.

The continuous development of Internet of Things technology has made it widely used in transportation, medical, security, and other fields. In the intelligent cold chain system, the Internet of Things technology combines freshness sensors, GPS (Global Positioning System) and wireless communication to achieve real-time monitoring of the entire cold chains. As shown in Figure 4, when the temperature of the refrigerated vehicle exceeds the set range, the temperature warning system will be triggered and send a signal to the refrigeration system, which will adjust the temperature and maintain the ambient temperature within an appropriate range [69]. The information collection methods of traditional cold chain systems mainly rely on manual recording or barcode scanning. The collection, transmission, processing, and sharing ability of the whole chain information are relatively poor, which means that they cannot respond to changes in environmental factors such as temperature and humidity in a timely manner during the distribution and transportation process [70]. The application of the Internet of Things technology in the cold chain system improves the intelligence of the cold chain system, enables real-time monitoring, tracking, and management of the whole chain, and greatly improves the integrity, transparency, security, and traceability of the cold chain systems. Mejjaouli et al. utilized RFID, temperature, humidity, and other sensors combined with GPS devices to conduct real-time monitoring of perishable food (meat and meat products). The sensor equipment sensed changes in external environmental conditions and monitored perishable food in real time through a wireless sensor network. If the food quality changes, the transportation route can be changed or the food can be sold nearby according to the actual quality and requirements, so as to reduce the economic losses caused by food decay [71]. Similarly, this method also has a wide application prospect in fruit and vegetable transportation. On the basis of ensuring the quality of fruits and vegetables and reducing their loss and waste, it can also reduce the need for manual participation and improve the overall integrity and intelligence of the cold chain system.

RFID, GPS, wireless communication, and other IOT sensing devices carry out real-time monitoring of the entire cold chain system and transmit various parameter data such as product information and environmental factors in real time. This enables real-time information sharing between cold chain nodes, and provides corresponding decision support for manufacturers, operators, regulators, and consumers. Additionally, this technology solves some problems in the cold chain system, such as information lag and temperature overruns that affect the efficiency and integrity of the cold chain to a certain extent, and effectively guarantees the quality and safety of transportation products. However, at the same time, the system has high configuration costs and high energy consumption, greatly increasing the cost of the cold chain system, prolonging the economic return cycle of enterprises, and reducing the economic efficiency of related enterprises. This is also the main reason for the low utilization of IOT technology in the food supply chains. Currently, researchers are improving the manufacturing process of sensors to reduce the cost of individual sensing devices, or improving information sensing technologies and optimizing supply chain systems to reduce the number of sensor devices used, thereby solving the problem of high cost in cold chain systems. For example, Fang et al. optimized and upgraded RFID tags. They allowed for simultaneous monitoring of product inventory, environmental temperature, ambient humidity, and other parameters, and effectively improved the efficiency of RFID tags in the cold chain system and reduced the number of RFID tags used, thus reducing the cost of the cold chain system [72].

In addition, the cold chain system also integrates a large amount of private information, which may cause significant losses to stakeholders if lost or stolen. Therefore, ensuring the privacy and security of the cold chain systems’ data is another focus in current and future research.

### 4.2. Traceability Based on Blockchain Technology

Blockchain is a distributed ledger technology. Due to its unique intelligent contract and consensus mechanism, blockchain has the characteristics of decentralization, low cost, high efficiency, traceability, security, and reliability. Blockchain technology can guarantee the integrity, transparency, and security of all transactions and data [73,74]. Blockchain technology supports the distributed consensus mechanism, which means creating immutable transaction records in the public ledger, allowing all participants to know every event and transaction, thereby establishing the trust relationship between entities [74]. Blockchain technology was first applied to bitcoin encryption to maintain transactions and avoid double expenditure, then gradually expanded to finance, securities, insurance, food, and other fields [75]. When the cold chain systems use RFID tags, GPS, and wireless communication technology to monitor the chain, although they can provide valuable information for real-time monitoring and traceability of food quality, the client–server mode of the system has problems with centralization and tamperability of data. Additionally, the accuracy and security of the data cannot be guaranteed. These adversely affect the supervision of cold chain systems and food quality and safety, which significantly increases the demand of producers, operators, regulators, and consumers for food traceability and safety [76,77]. In addition to ensuring the quality of food, blockchain technology can also ensure the validity and authenticity of data. When the product information, real-time location, ambient temperature, environmental humidity, and other parameters of each link of the cold chains are added to the blockchain systems, they will be permanently stored and cannot be tampered with. For example, Azzi et al. [74] utilized the unique smart contract and consensus mechanism of blockchain to integrate the intelligent cold chain systems with blockchain technology, thereby improving the security and reliability of data at all stages of the chain.

Blockchain technology connects all participants throughout the whole industrial chain from the origin to consumers, breaks the information barriers between all links, which achieves high-speed data transmission through the Internet. Additionally, it ensures the authenticity and effectiveness of data through smart contract technology and consensus mechanisms. It puts forward effective strategies to solve problems, such as privacy protection, data falsification, and information tampering [78]. Producers, operators, regulators, and consumers can obtain food information in real time through data query, which greatly improves the traceability and safety of cold chain products. Torky et al. [79] used RFID, WSN, near-field communication, compressed sensing, and other information technologies to collect and transmit food-related information, and verified the acquired data through blockchain to ensure the accuracy and integrity of data collection.

Although the tamper-resistant feature of blockchain ensures data security in the cold chain system, the system has poor fault tolerance and requires an accurate data input system [78]. As for the food preservation cold chain systems, the life cycle of food is long, the chain from the origin to the consumer is too long, the information varies greatly, and the scene is complex, so it is difficult to express the situation in code, and there are contradictions and conflicts between the data privacy and transparency in the blockchain system. Therefore, the government and relevant regulatory agencies should formulate different standards and agreements for different products, and develop corresponding laws and regulations so as to ensure the minimum business standards, the rationality and security of data disclosure, which could reduce the information asymmetry between the two sides of the transaction, and improve the work efficiency of the supply chain [80].

Powell et al. developed the Beef Ledger platform, which combines blockchain technology with Internet of Things technology to store and encrypt data across the entire beef supply chain. Staff from producers to consumers can query historical beef data through the platform, but the data cannot be tampered with, which ensures the authenticity and traceability of data [81].

### 4.3. Traceability Based on Digital Twinning Technology

In order to improve and optimize the efficiency of intelligent cold chain systems and minimize the impact of the cold chain environment on food quality, it is necessary to strictly analyze the corruption mechanism of food and the change in environmental parameters throughout the process of food production and circulation. This means that different food categories require different regulation of environmental parameters. In practical life, testing different food groups may cause a waste of resources and energy. Through digital twin technology, researchers can effectively realize efficient digital modeling and the simulation of cold chain systems, establish a virtual cold chain, and then conduct digital simulation and analysis of the food corruption mechanism. By combining the life cycle of food with the virtual cold chains for comprehensive evaluation, the cold chain systems can be optimized under the premise of effectively reducing energy consumption. This method can reduce food consumption, and the storage and transportation efficiency of the supply chain can be improved with this method [82,83,84].

Digital twin technology is an innovative technology derived from the concept of simulation. It realizes real-time mapping, interaction, integration, and data fusion between a physical entity and digital models by virtualizing and digitizing the physical entity and establishing mathematical models related to it. The real-time monitoring, diagnosis and prediction of a physical entities’ state can be realized through the technologies of situation simulation, actual testing, and data processing, so as to regulate and optimize the entities [82,84]. Digital twin technology organically combines physical entities with a virtual model, simulates the whole process of the physical entities through sensor equipment, and returns data to the central server for storage and processing. On this basis, the whole processes of physical entities are regulated and optimized. Researchers have applied it to intelligent cold chain systems by establishing a virtual cold chain to simulate and regulate the whole cold chain logistics systems of fruits, vegetables, and meat products, so as to solve the problems of the high cost of cold chain systems and unstable product quality. For example, Agalianos et al. established a virtual model of meat products through computational fluid dynamics (CFD), and simulated its supply chain entity before practical application so as to optimize and regulate its industrial processes [85].

Coimbra et al. adopted digital twining technology based on CFD to digitize the broiler carcass precooling process, which could effectively predict the temperature distribution inside the carcass, so as to optimize the precooling condition configuration, improve the precooling efficiency, shorten the precooling time, and reduce energy consumption [86]. Merai et al. utilized CFD to model and simulate the cooling process of a pork carcass to explore the internal temperature distribution. The results showed that properly raising the initial temperature of pork carcass during transportation can shorten the entire precooling time in the cold chain system by nearly 10 h, which can reduce the energy consumption and cost of precooling, and realize the optimization and regulation of the meat supply chain [87]. Wu et al. combined the life cycle assessment with the virtual cold chain. They established a virtual cold chain and used computational fluid dynamics (CFD) to track and monitor the thermal history and mass attenuation of the citrus cooling process in the entire cold chain system. Then, they evaluated the citrus quality related to the cooling process and provided information on the temperature-dependence of citrus quality evolution. This paper explored the changing relationship between air vent design and citrus quality, and contributed new strategies and ideas for the combination of whole life cycle evaluation and virtual cold chain, which is conducive to the selection of the optimal scheme for citrus cold chain logistics. In addition, it is helpful for the regulation and optimization of logistics energy consumption in the cold chain [88]. As a fruit with a short shelf life, strawberries are easily affected by mechanical damage, microbial decay, and water loss, and thus decrease in value, resulting in a certain amount of waste and economic losses. As a result, strawberries require very strict temperature management. With proper temperature control, microbial activity, water loss, and strawberry respiration processes can be reduced. Nalbandi et al. used experimental and modeling methods to design new packaging and cooling systems for strawberry precooling. The CFD model was used to visualize the airflow path and distribution in depth, thus achieving faster and more uniform cooling of strawberries [89]. Han et al. simulated the heat and mass transfer characteristics of fresh tomatoes by establishing a computational fluid dynamics model. In doing so, they dynamically predicted the spatio-temporal distribution of temperature and water content of packaged tomatoes in the whole cold chain, and quantitatively evaluated the cold chain break (CCB) parameters (such as temperature, relative humidity, effect of airflow rate, and duration) on temperature fluctuations and tomato mass loss. Research has shown that reducing the temperature of CCB and increasing the relative humidity are the best methods to reduce the temperature fluctuation and water loss of tomatoes under the CCB scenario, followed by reducing the duration of CCB and airflow rate [90].

Mukama et al. used CFD simulation to improve the vent design of the existing commercial carton of pomegranates, and reconstructed the vent of the carton, minimizing the airflow path obstruction, which significantly improved the cooling rate and cooling uniformity [91]. Hoang et al. modeled the temperature distribution of chickens in the precooling process through CFD and explored the influencing factors of various parameters in the precooling process on chicken quality so as to achieve the regulation of the precooling process [92].

Verboven et al. pointed out that, in the context of industrial digitization and the emergence of the Internet of Things, multi-scale, multi-phase, and multi-physical field-coupled models based on CFD digital twinning technology improve the process and design of the food supply chain, rather than being limited to a specific part or scale of the process. Through the simulation of the whole food supply chain, the exploration of food corruption mechanisms, the optimization of the food supply chain, and the improvement of cold chain logistics, food quality and shelf life are maximized, energy utilization efficiency is enhanced, and economic losses are reduced [82].

Therefore, cold chain models based on digital twinning technology enable researchers to carry out complex conceptual designs and analyses of the whole food supply chain, so as to regulate and optimize each link. This technology saves the experiment time and cost, and has important practical significance for improving food quality control efficiency and cold chain system optimization [93]. Table 2 shows the application of a smart cold chain.

## 5. Outlook and Challenges

The intelligent cold chain systems connect regulators, producers, and consumers, provide them with dynamic environmental parameter control, fault warning, real-time information sharing, real-time display of food quality and shelf life, real-time traceability of product locations, and other information throughout the supply chain. Therefore, China attaches great importance to the development of intelligent cold chain systems and has introduced relevant policies to support their development, so as to improve the efficiency and benefit of the industry. In 2020, a document issued by the Central Committee said that China should increase the construction of cold chain system modernization and intelligent system construction, and accelerate the construction of modern cold chain system industrial parks. Other countries have also introduced relevant policies to vigorously develop the cold chain industry. However, there is still a lack of standards regarding cold chains throughout the world, and the globalization of cold chain system standards still has a long way to go.

The rapid development of Internet of Things technologies has facilitated wireless real-time environmental parameter monitoring in the supply chain of fresh fruits and vegetables. This increases the possibility of reducing the amount of product lost and wasted in the supply chain. Monitoring systems based on the Internet of Things can be used to gain insight into current product status, perform weakness analysis on parameter profiles, and strengthen process control.

The interruption of any link in the intelligent cold chain system (such as temperature interruption) will accelerate the deterioration of food and shorten the shelf life of food. At present, the intelligent cold chain systems have problems such as poor stability, high error rate, poor dynamic perception of product quality, slow cold chain data transmission and processing speed, and lacking the unified management standards and laws and regulations. The rapid development of the Internet of Things effectively improves the real-time monitoring efficiency of parameters such as environmental temperature and humidity in the intelligent cold chain system of fruits and vegetables. Moreover, it can analyze the weakness of the parameter configuration file and strengthen the process control, which greatly improves the reduction of food loss and waste in each link. In addition, the combination of Internet of Things technology and digital simulation helps to optimize the parameters of storage and logistics chains [99]. For example, the goal of reducing product waste and loss can be achieved by applying FEFO (first past expiration, first out) rather than the traditional FIFO (first in, first out) principle in smart warehousing [100].

Multiple sensor devices, data compatibility, interoperability, and the standards related to data formats, data exchange, and technical equipment are major challenges for IoT-based systems [101]. The existence of blockchain technology solves these problems. Through untampered and shared data, participants use consistent standards to realize information sharing and traceability among multiple devices, increase the collaboration among participants of all links, and improve the overall efficiency of the cold chain system [102,103,104,105].

With the continuous development and deep integration of computer technology and artificial intelligence, intelligent cold chain systems are becoming more digital, intelligent, and efficient, which is also the main direction of innovation and optimization of intelligent cold chain systems in the future [106]. Secondly, while developing the intelligent cold chain system, researchers should update the components of the intelligent cold chain systems. While improving the performance, they should reduce the cost of the components, and actively promote the large-scale application of the cold chain system across the country.

## 6. Conclusions

This article provides a comprehensive overview of the research status, shortcomings, future trends, and challenges of cold chain systems from the perspectives of traditional preservation technologies, intelligent cold chain system components, and system regulation and optimization. This is meaningful for improving the efficiency of the cold chain system, improving the integrity of the cold chain system, ensuring product quality and safety, and reducing food waste and loss.

The rapid development of computer technology provides new ideas and innovation points for the preservation of fruits and vegetables. At present, there are supply chains for specific fruits and vegetables, but the combination of cold chain systems and communication technologies lacks simulation for general fruits and vegetables, and also lacks specific standards. In the future, the degree of integration of cold chain systems and artificial intelligence should be increased to promote cold chain systems towards the direction of low energy consumption, low cost, high preservation, and high intelligence. At the same time, governments at all levels should formulate corresponding unified standards, and producers and operators at all levels should strictly implement and abide by rules. This can establish a truly transparent cold chain system with consumers, establish trust relationships between entities, which can improve the efficiency of existing cold chain systems, and truly achieve resource-saving and sustainable smart agriculture.

## Figures and Tables

**Table 1:** Application of nano antibacterial agents in food preservation.

Antibacterial Agent	Food Packaging	Food	Antimicrobial Action	References
Ag NPs	Chitosan	Lychee	*S. aureus, E. coli*	[28]
	PE	Carrot	*S. Typhimurium and S.aureus*	[29]
	PVC	Papaya	*E. coli and L.monocytogenes*	[30]
	Chitosan	Strawberry	*E. coli,*	[31]
	Chitosan-starch	Peach	*S. aureus, E. coli, and C.albicans*	[32]
	Cellulose	Tomatoes	*S. aureus, E. coli*	[33]
Se-Ag NPs	Gelatin	Mini kiwis	*S. aureus, E. coli*	[34]
Au NPs	PVA	Banana	*E. coli*	[35]
	Cellulose/keratin	--	*S. aureus, Enterococcus (VRE)*	[36]
	Cellulose	--	*E. coli*	[37]
TiO_2_ NPs	k-carrageenan and KGM	Strawberries	*Penicillium*	[27]
	Chitosan	Potato	*E. coli*	[31]
	PBAT-starch	Banana	*E. coli*	[38]
ZnO NPs	Chitosan and cellulose acetate phthalate	Black grapes	*S. aureus, E. coli,*	[32]
	Chitosan	Tomato	*Alicyclobacillus acidoterrestris, S.aureus, E.coli, Salmonella*	[39]
	--	Green soy bean	*coliforms, yeast, molds*	[40]
	--	Pomegranate	*yeast , mold*	[41]

**Table 2:** Application of a smart cold chain.

Method	Food	Research Results	References
Digital twinning technology	Strawberry	Presents a novel, holistic approach to selecting the optimal ventilated packaging of strawberries from farm to retailer. This approach can help reduce food loss and contribute towards making supply chains smart and efficient.	[94]
Kinetically modelled, enzymatic TTIs	Ready-to-eat chilled smoked fish	Appropriate TTIs were selected for shelf life monitoring of smoked fish in the cold chain.	[95]
Blockchain technology	Livestock products	Real-time risk point detection for food safety can reduce food fraud and contamination while also strengthening the mechanism for recalling affected batches of products.	[96]
Proportion-integral-derivative (PID) control algorithm, 4G	Aquatic products	Established a mathematical model for the system based on the step response method, realized the gas dynamic volumetric based on the embedded PID control algorithm and mass flow controller; used 4G network for remote data transmission; realized remote monitoring function of mobile client through cell phone terminal applet software.	[97]
A diffusion-based time–temperature-indicator (TTI), kinetically modelled.	Juice	TTI systems are efficient and economical tools for monitoring, recording, and translating the overall effect of temperature history on food safety and quality at a product unit level.	[98]

## Data Availability

The authors confirm that the data supporting the findings of this study are available within the article.
